# Modeling the Role of Wnt Signaling in Human and *Drosophila* Stem Cells

**DOI:** 10.3390/genes9020101

**Published:** 2018-02-16

**Authors:** Prameet Kaur, Helen Jingshu Jin, Jay B Lusk, Nicholas S. Tolwinski

**Affiliations:** 1 Yale-NUS College Research Labs, Singapore 117608, Singapore; prameet.kaur@yale-nus.edu.sg (P.K.); jingshu.jin@u.yale-nus.edu.sg (H.J.J.); jay.lusk@u.yale-nus.edu.sg (J.B.L.); 2 Department of Biological Sciences, National University of Singapore, Singapore 117543, Singapore

**Keywords:** Wnt, stem cells, *Drosophila*, induced pluripotent stem cells

## Abstract

The discovery of induced pluripotent stem (iPS) cells, barely more than a decade ago, dramatically transformed the study of stem cells and introduced a completely new way to approach many human health concerns. Although advances have pushed the field forward, human application remains some years away, in part due to the need for an in-depth mechanistic understanding. The role of Wnts in stem cells predates the discovery of iPS cells with Wnts established as major pluripotency promoting factors. Most work to date has been done using mouse and tissue culture models and few attempts have been made in other model organisms, but the recent combination of clustered regularly interspaced short palindromic repeats (CRISPR) gene editing with iPS cell technology provides a perfect avenue for exploring iPS cells in model organisms. *Drosophila* is an ideal organism for such studies, but fly iPS cells have not yet been made. In this opinion article, we draw parallels between Wnt signaling in human and *Drosophila* stem cell systems, propose ways to obtain *Drosophila* iPS cells, and suggest ways to exploit the versatility of the *Drosophila* system for future stem cell studies.

## 1. Stem Cells in Human and *Drosophila*

Stem cells have the ability to self-renew and to produce specialized cells during development, normal organ function, and in response to tissue damage. Recent studies have shown the importance of the highly conserved Wnt signaling pathway in controlling the cell renewal process in various organisms ranging from humans to mice and flies [1,2]. The groundbreaking discovery that the expression of POU domain, class 5, transcription factor 1 (OCT4), sex determining region Y (SRY), transcription factor SOX-2 (SOX2), Kruppel-like factor 4 (KLF4), and myelocytomatosis oncogene (C-MYC) (also known as the Yamanaka factors) was able to induce pluripotency in differentiated cells meant that the seemingly unidirectional differentiation process could be reversed [3]. These factors have proven to be effective in human cells and mouse systems, but their application to *Drosophila melanogaster*, the ideal organism for rapid genetic studies, has not been explored adequately [4].

## 2. Wnt Signaling in Stem Cells

Wnt signaling refers to a collection of intracellular signal transduction pathways that result in a variety of cellular and developmental outcomes. First discovered as insertion sites for mouse mammary tumor virus [5] and patterning genes in *Drosophila* [6,7], the cloning of both fly and mouse *Wnt1* showed just how well conserved signaling is and the close relationship between development, stem cells, and cancer [8,9,10]. The best studied of the Wnt pathways, or the so-called canonical signaling pathway, transduces the signal received from extracellular Wnt ligand binding by stabilizing the cytoplasmic pool of β-Catenin protein, leading to its nuclear translocation and transcriptional activation through the transcription factor (TCF) proteins [11]. In contrast, the non-canonical Wnt pathways function through cytoplasmic and plasma membrane proteins to affect various cell polarity roles [12]. Whether there is a role for non-canonical Wnt signaling in stem cells is not known, but canonical Wnt plays a role in stem cell maintenance and in establishing pluripotency. Wnt regulates various types of stem cells, especially through its role as a niche factor to maintain self-renewal [13,14]. These include neural stem cells, intestinal cells, hair follicles, mammary gland cells, and hematopoietic cells (Table 1). The converse of *healthy* stem cells are cancer cells, which retain the ability to self-renew but instead of regenerating specific organs, generate abnormal growths which are all hallmarks of Wnt-related cancers [15,16]. 

The first evidence of the importance of Wnt in adult stem cell biology was observed when genetic disruption of mouse transcription factor 4 (*Tcf4)* resulted in loss of intestinal stem cells [17], followed soon after by the discovery of Wnt’s role in hematopoietic stem cells [18,19]. Thereafter, several Wnt pathway components have proven to be essential for several types of stem cells. For example, Sato and et al. showed that activating the Wnt/β-Catenin pathway with wingless-type MMTV integration site family, member 3A (Wnt3a)-conditioned media or with a glycogen synthase kinase 3 (GSK3) inhibitor could stimulate embryonic stem cell self-renewal [20]. This work was extended by Silva et al. who showed that pluripotency could be induced by using media containing 2 kinase inhibitors (2i Media), with the first inhibiting the negative regulator of Wnt signaling GSK3, and the second inhibiting the mitogen-activated protein kinase/extracellular regulated kinase (MEK) signaling [21]. Wnt signals were further shown to be self-renewal factors for mouse embryonic stem cells by preventing epigenetic changes that cause differentiation into the developmentally more advanced epiblast stem cells (EpiSC) [22]. Wnt regulates this transition with high Wnt activity in naïve/mouse embryonic stem cells (ESCs) and low Wnt activity in primed (EpiSCs and human ESCs) stem cells, leading to different outcomes depending on the state of the pluripotent stem cells. In naïve cells, Wnt promotes self-renewal, whereas in primed cells, Wnt drives differentiation [22]. The importance of Wnt signaling in stem cell biology is reiterated in various cell types such as epidermal cells and hair follicles [23,24,25], mammary glands [26], and the hematopoietic system [18,19] (Table 1).

**Figure 1:**
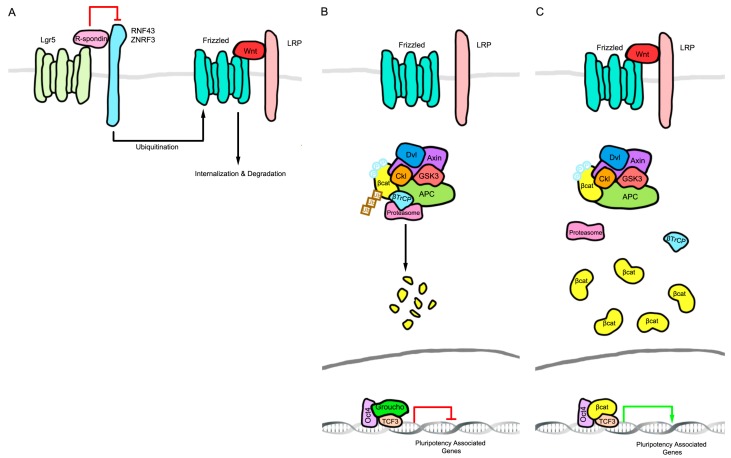
Schematic of the Wnt signaling pathway that determines stem cell self-renewal. (**A**) Leucine-rich repeat-containing G-protein coupled receptor 5 (LGR5) binds to R-spondin proteins, which in turn prevents the endocytosis of Wnt/Receptor complexes through ring finger 43 (RNF43)/E3 ubiquitin ligase zinc and ring finger 3 (ZNRF3)-mediated membrane clearance. This maintains the Wnt-bound receptor complex (Wnt/Frizzled/ LDL receptor related protein (LRP)) at the plasma membrane, nucleating activation complexes and inhibiting β-Catenin degradation enhancing Wnt signaling; (**B**) In the absence of Wnt protein, β-Catenin is degraded and transcription factor 3 (TCF3)-mediated repression of POU domain, class 5, transcription factor 1 (*OCT4*), sex determining region Y (*SRY*) transcription factor SOX-2 (*SOX2*), and Nanog homeobox (*NANOG*) target genes prevents self-renewal. (**C**) Stabilized β-Catenin upon the activation of Wnt signaling suppresses TCF3-mediated repression of *OCT4*, *SOX2*, and *NANOG* target genes to mediate signaling for self-renewal. Dvl: Dishevelled; APC: Adenomatosis polyposis coli; Ckl: Casein kinase 1; βCat: β-Catenin; βTrCP: β-transducin repeat containing E3 ubiquitin protein ligase; Ub: Ubiquitination.

The Wnt pathway components leucine-rich repeat-containing G-protein coupled receptor 5 (LGR5) and AXIN 2 serve as indicators of cells responsive to Wnt signaling and hence undergoing tissue renewal in various organs [26,27]. G-protein coupled receptor 5 affects Wnt signaling by binding to R-spondin proteins, which in turn prevent the endocytosis of Wnt/Receptor complexes through ring finger 43 (RNF43)/E3 ubiquitin ligase zinc and ring finger 3 (ZNRF3) mediated membrane clearance (Figure 1A). This maintains the Wnt-bound receptor complex (Wnt/Frizzled/ LDL receptor related protein (LRP)) at the plasma membrane, nucleating activation complexes and inhibiting β-Catenin degradation, thus enhancing Wnt signaling [28,29]. AXIN2 is a direct transcriptional target for β-Catenin/TCF and its transcription has been exploited as an indicator of Wnt pathway-responsive cells [26,30]. In addition to marking stem cells through AXIN2 and LGR5 expression, β-Catenin activity is required for self-renewal under either 2 kinase inhibitors (2i) or leukemia inhibitory factor (LIF) + GSK3-inhibitor conditions, showing that β-Catenin-dependent signaling is a downstream effector of GSK3 inhibition [31,32]. β-Catenin then suppresses TCF3-mediated repression of *OCT4*, *SOX2*, and Nanog homeobox (*NANOG*) target genes to mediate signaling for self-renewal [32,33,34,35,36,37,38,39,40,41]. The downstream mechanism of β-Catenin-mediated self-renewal has, however, been controversial. A TCF-independent mechanism for Wnt-β-Catenin-mediated mESC self-renewal has also been proposed whereby a direct interaction between β-Catenin and OCT4 transcriptionally activates OCT4-bound self-renewal genes (Figure 1B,C) [42,43,44].

In addition to stimulating self-renewal in ESCs, Wnt signaling prevents differentiation. Wnt signaling inactivates the destruction complex (composed of casein kinase (CK1), GSK3, AXIN, and adenomatous polyposis coli (APC)), which marks β-Catenin for ubiquitin-mediated degradation. With the destruction complex off, free β-Catenin translocates to the nucleus to transcribe target genes. When ESC were made with APC mutations that activated Wnt signaling by increasing β-Catenin levels, these cells were unable to differentiate into embryonic layers [45]. A similar result was observed in ES cells lacking both GSK3α and β isoforms [46]. 

## 3. Wnt Signaling and Stem Cells in *Drosophila*

**Figure 2:**
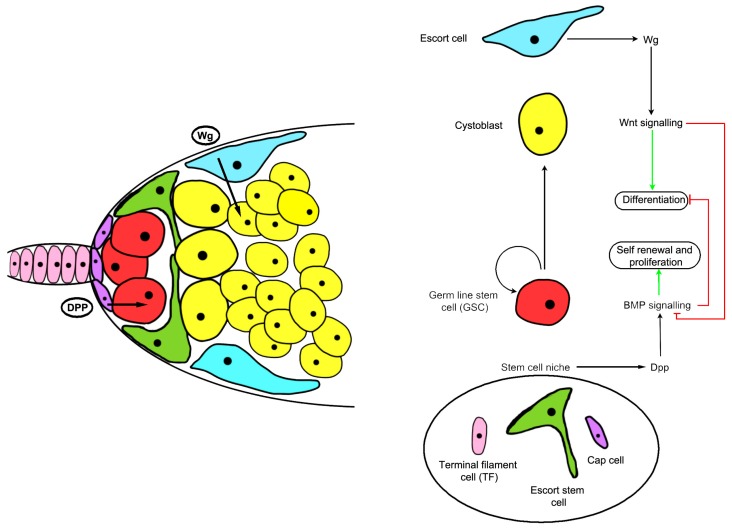
Structure and signaling mechanisms of the *Drosophila* ovarian germline stem cell (GSC) niche. Cap cells (purple) and escort stem cells (green) function as a niche to maintain GSCs (red), allowing germ cells outside the niche to differentiate. A schematic diagram showing that wingless (Wg) signals control inner germarium sheath cells (IGC) maintenance and promote germ cell differentiation by preventing bone morphogenetic protein (BMP) signaling. Dpp: Decapentaplegic.

In *Drosophila*, the major systems for addressing stem cell behavior have been germline stem cells and intestinal stem cells [54,55]. In the germline, the *Wnt* homologous gene *wingless (wg)* is required for stem cell maintenance. Disruption of canonical Wnt signaling in the inner germarial sheath cells (or escort cells) that surround the germline stem cells (Figure 2) results in increased decapentaplegic (dpp) mRNA expression, expansion of germline stem-like cells, and increased bone morphogenetic protein (BMP) responsiveness in the germline (Figure 2) [56,57]. Wnt signaling in the stem cell niche is increased with age in flies while BMP signaling is reduced, signifying the importance of Wnt signaling-mediated cell-cell communication to modulate niche stem cell signaling [57]. Another intriguing finding in the germline stem cell niche was that asymmetric cell division was regulated by Wnt components, suggesting a possible non-canonical signaling role in stem cells [58]. These non-canonical signaling pathways which influence apico-basal polarity have not been studied in great detail, especially as they relate to stem cells, but *Drosophila* stem cells and development show some possible avenues to explore [59,60,61]. 

**Figure 3:**
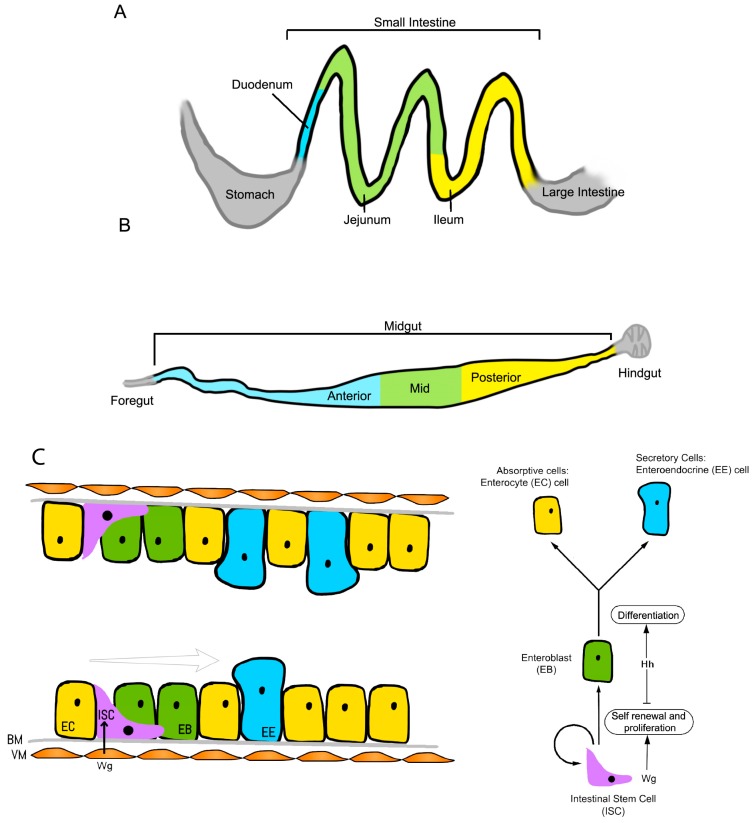
Schematic of the (**A**) mouse and (**B**) *Drosophila* intestinal systems; (**C**) Development of intestinal stem cells in the *Drosophila* hindgut. Wg/Wnt1 secretion from the visceral muscle (VM) promotes intestinal stem cell (ISC) proliferation and self-renewal. As the cells divide and move away from the Wg signal, exposure to hedgehog (Hh) induces the onset of enteroblast (EB) differentiation. Notch signaling determines the cell fate, with strong notch signals in the EBs favoring differentiation into enterocytes (ECs). BM: basement membrane; EE: Enteroendocrine.

The *Drosophila* model has also proven to be crucial in establishing how intestinal stem cells are maintained in their self-renewing stem cell state. Takashima et al. showed that the intestinal stem cells of the posterior intestine are localized to an anterior narrow region, and these are under the control of local *Wnt1* and hedgehog (*hh*) signals [62]. Wnt proteins, like Hh proteins, are lipid-modified, which may constrain them to act as short-range signals [63]. This restricted expression of Wnt in the anterior region serves as a niche signal that maintains cells in a self-renewing mode. Moving away from the Wnt source, cells divide and move posteriorly as well as proliferate rapidly, during which hedgehog signaling takes over to allow cell cycle exit and differentiation. Finally, notch signaling results in differentiation into enterocyte cells (Figure 3) [62]. Additionally, recent work showed that adenoma-like structures form in response to Wnt activation in the fly intestine, recapitulating mammalian experiments [64,65], thus the *Drosophila* model provides insights into the spatial control and regulation of stem cell self-renewal.

## 4. Benefits of Using *Drosophila* as a Model System

*Drosophila melanogaster* is a strong model organism for research into stem cell biology, possibly including pluripotency. *Drosophila* presents opportunities for powerful genetic manipulation; the ability to make straightforward, high-fidelity knock-outs, knock-ins, and knock-downs, allowing for great versatility in manipulating genes [66,67]. Additionally, modern imaging methods such as light-sheet microscopy and confocal microscopy allow for precise in vivo observation. These, when combined with optogenetic approaches perturbing signal transduction pathways, could translate into an in vivo 2i system, where pathways could be turned on or off and the effects could be imaged [68,69,70,71,72]. In mice, pluripotency factors have been demonstrated to be functionally redundant; therefore, given the relative difficulty of manipulating mice genetically, *Drosophila* may be an effective system to dissect the individual influences of each gene more carefully as related to Wnt signaling [38,73]. Transcriptional profiling approaches have shown some similarities between Wnt in human stem cells and fly cells, such as the gene Sp5 transcription factor (*Sp5)* [74,75,76].

Pluripotency genes have not yet been extensively characterized in *Drosophila*, although various populations of cells including the adult posterior midgut, embryonic nervous system, and germline have been found to develop from stem cell-like progenitors [77,78,79]. Recently, Rosello et al. demonstrated that mouse pluripotency factors could induce colony formation in *Drosophila* S2 cells as well as express a handful of endogenous *Drosophila* adult stem cell markers, indicating the ability to generate an iPS-like phenotype [4]. However, the ability of these iPS-like cells to form teratomas and chimeric embryos, rigorous tests of pluripotency in vivo, were not carried out. While the S2 cells did not become iPS cells, they attained many of the characteristics of iPS cells, indicating that these factors may have significant utility in the *Drosophila* system for investigating stem-cell and stem-cell-like behavior. S2 cells are commonly used, but these experiments are fundamentally limited as S2 cells are secondary cell lines. Instead, primary cell lines and in vivo models should be more effective in producing results that are more relevant to organisms. Ideally, fly homologs of the Yamanaka factors should be used. Currently, the fly genes are hypothesized to be *myc*, *luna*, *nubbin*, and *sox21a* [80]. Once effective homologs are found which induce pluripotency, the fly system can be utilized as a cheap, fast, and accurate way to probe interactions between signaling and pluripotency. Additionally, undertaking in vivo work with *Drosophila* allows for the ability to utilize the broad range of molecular techniques in use in the organism. For example, one could utilize CRISPR/Cas9 approaches to selectively activate or inactivate homologs of the Yamanaka factors in specific tissue regions during specific stages of development. 

**Figure 4:**
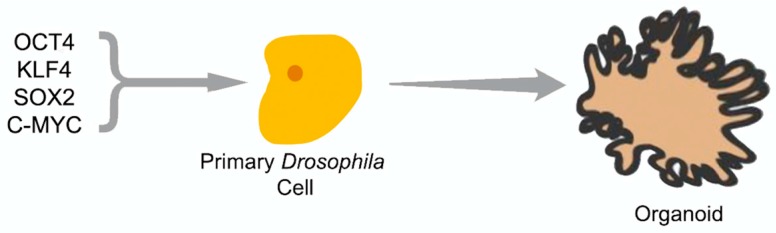
Experimental procedure: Transformation of primary *Drosophila* cells with the four stem-cell genes from humans: *OCT4*, *KLF4*, *SOX2*, and *C-MYC*. The cells are expected to develop induced pluripotent stem (iPS) cell-like characteristics in media containing 2 kinase inhibitors (2i media) and grow into an organoid once differentiation factors are applied or 2i media is removed.

A major reason to undertake in vivo studies with *Drosophila* iPS cells is that, recently, Ocampo et al. showed that the systemic expression of pluripotency factors OCT4, SOX2, KLF4, and c-MYC (OSKM) could induce partial reprogramming in vivo in mice [81,82,83]. These authors observed an amelioration of cellular markers of aging and improved treatment of metabolic disease and muscle injury. These studies then provide a precedent that OSKM pluripotency factors can effectively induce a pluripotent system in primary culture and in vivo. Therefore, it should be possible to utilize this approach to create reprogrammed *Drosophila* organoids which could be utilized for cancer or aging studies (Figure 4), or to use adult flies to study the induction of stem cells in adults. Insect cell organoids could be made from a variety of mutant or engineered fly strains and would take advantage of the smaller genome containing fewer redundant or paralogous genes with overlapping functions. This system could then be used to test various Wnt signaling agonists and antagonists for their ability to stimulate stem cells, effectively creating an in vivo system for screens and possibly combining Wnt’s role in stem cells with its role in aging [84].

## Figures and Tables

**Table 1:** Role of Wnt signaling in different cell types.

Wnt Signaling in Stem Cells			
Organ	Cell Type	Wnt Function	References
Embryo	Mouse embryonic stem cells	- Wnt pathway regulates transitions from ESCs to EpiSCs. - Wnt3a protein with LIF is sufficient to support ESC self-renewal and the derivation of new ESC lines through canonical β-Catenin signaling.	[22,47,48]
Nervous system	Mouse brain-derived neural stem cells	- Dual inhibition of the mitogen-activated protein kinase signalling (MEK/ERK) pathway and of glycogen synthase kinase-3 (GSK3) (a negative regulator of Wnt signaling), along with exposure to LIF, induces full reprogramming to induced pluripotent stem (iPS) cells. - Wnt3a/β-Catenin signaling is responsible for neurogenesis in the adult hippocampus and cerebral cortex.	[21,49,50]
Skin	Mouse hair follicle stem cells	- Autocrine Wnt signaling maintains hair follicle stem cell potency, while the ectopic expression of DKK prevents the development of all types of hair follicles, as well as tooth and mammary gland development.	[25,30]
Hematopoiesis	Mouse hematopoietic stem cells	- Ectopic expression of Axin and the Frizzled cysteine-rich domain leads to reduced stem cell growth, while the activation of Wnt signaling increases the expression of hematopoietic stem cell self-renewal genes, *HoxB4* and *Notch1.*	[18,19]
Mammary Gland	Mouse mammary stem cells	- Exposure to Wnt3a protein greatly increases the clonogenicity of mammary stem cells while retaining their full developmental potential in vivo. - Protein C receptor (*Procr*), a novel Wnt target in the mammary gland, is a marker for a unique population of multipotent mouse mammary stem cells.	[51,52]
Intestine	Mouse colorectal cancer cells	- Disruption of β-Catenin/transcription factor 4 (TCF-4) activity in colorectal cancer cells blocks the proliferative program of colon crypts and induces a differential program. - LGR5, a Wnt target gene as well as Wnt signal enhancer, serves as a marker for stem cells.	[17,27]
Liver	Mouse hepatocytes	- Damage-induced *Lgr5*^+^ stem cells generate hepatocytes and bile ducts in vivo.	[53]

ESC: embryonic stem cell; EpiSC: epiblast-derived stem cell; LIF: leukemia inhibitory factor; DKK: dickkopf Wnt signaling pathway inhibitor; LGR5: leucine-rich repeat-containing G-protein coupled receptor 5; Wnt3a: wingless-type MMTV integration site family, member 3A.

## References

[1] Li H., Jasper H. (2016). Gastrointestinal stem cells in health and disease: From flies to humans. Dis. Models Mech..

[2] Casali A., Batlle E. (2009). Intestinal stem cells in mammals and *Drosophila*. Cell Stem Cell.

[3] Takahashi K., Yamanaka S. (2006). Induction of pluripotent stem cells from mouse embryonic and adult fibroblast cultures by defined factors. Cell.

[4] Rossello R.A., Chen C.C., Dai R., Howard J.T., Hochgeschwender U., Jarvis E.D. (2013). Mammalian genes induce partially reprogrammed pluripotent stem cells in non-mammalian vertebrate and invertebrate species. Elife.

[5] Nusse R., Varmus H.E. (1982). Many tumors induced by the mouse mammary tumor virus contain a provirus integrated in the same region of the host genome. Cell.

[6] Nusslein-Volhard C., Wieschaus E. (1980). Mutations affecting segment number and polarity in drosophila. Nature.

[7] Sharma R.P., Chopra V.L. (1976). Effect of the wingless (wg1) mutation on wing and haltere development in *Drosophila melanogaster*. Dev. Biol..

[8] Nusse R., van Ooyen A., Cox D., Fung Y.K., Varmus H. (1984). Mode of proviral activation of a putative mammary oncogene (*int-1*) on mouse chromosome 15. Nature.

[9] Baker N.E. (1987). Molecular cloning of sequences from *wingless*, a segment polarity gene in *Drosophila*: The spatial distribution of a transcript in embryos. EMBO J..

[10] Rijsewijk F., Schuermann M., Wagenaar E., Parren P., Weigel D., Nusse R. (1987). The *Drosophila* homolog of the mouse mammary oncogene *int-1* is identical to the segment polarity gene *wingless*. Cell.

[11] Van Amerongen R., Nusse R. (2009). Towards an integrated view of Wnt signaling in development. Development.

[12] Schlessinger K., Hall A., Tolwinski N. (2009). Wnt signaling pathways meet Rho GTPases. Genes Dev..

[13] Nusse R. (2008). Wnt signaling and stem cell control. Cell Res..

[14] Nusse R., Fuerer C., Ching W., Harnish K., Logan C., Zeng A., ten Berge D., Kalani Y. (2008). Wnt signaling and stem cell control. Cold Spring Harb. Symp. Quant. Biol..

[15] Jamieson C.H., Ailles L.E., Dylla S.J., Muijtjens M., Jones C., Zehnder J.L., Gotlib J., Li K., Manz M.G., Keating A. (2004). Granulocyte-macrophage progenitors as candidate leukemic stem cells in blast-crisis cml. N. Engl. J. Med..

[16] Reya T., Clevers H. (2005). Wnt signalling in stem cells and cancer. Nature.

[17] Korinek V., Barker N., Moerer P., van Donselaar E., Huls G., Peters P.J., Clevers H. (1998). Depletion of epithelial stem-cell compartments in the small intestine of mice lacking Tcf-4. Nat. Genet..

[18] Reya T., Duncan A.W., Ailles L., Domen J., Scherer D.C., Willert K., Hintz L., Nusse R., Weissman I.L. (2003). A role for Wnt signalling in self-renewal of haematopoietic stem cells. Nature.

[19] Willert K., Brown J.D., Danenberg E., Duncan A.W., Weissman I.L., Reya T., Yates J.R., Nusse R. (2003). Wnt proteins are lipid-modified and can act as stem cell growth factors. Nature.

[20] Sato N., Meijer L., Skaltsounis L., Greengard P., Brivanlou A.H. (2004). Maintenance of pluripotency in human and mouse embryonic stem cells through activation of wnt signaling by a pharmacological GSK-3-specific inhibitor. Nat. Med..

[21] Silva J., Barrandon O., Nichols J., Kawaguchi J., Theunissen T.W., Smith A. (2008). Promotion of reprogramming to ground state pluripotency by signal inhibition. PLoS Biol..

[22] Ten Berge D., Kurek D., Blauwkamp T., Koole W., Maas A., Eroglu E., Siu R.K., Nusse R. (2011). Embryonic stem cells require Wnt proteins to prevent differentiation to epiblast stem cells. Nat. Cell Biol..

[23] Lim X., Tan S.H., Yu K.L., Lim S.B., Nusse R. (2016). *Axin2* marks quiescent hair follicle bulge stem cells that are maintained by autocrine Wnt/β-Catenin signaling. Proc. Natl. Acad. Sci. USA.

[24] DasGupta R., Fuchs E. (1999). Multiple roles for activated LEF/TCF transcription complexes during hair follicle development and differentiation. Development.

[25] Andl T., Reddy S.T., Gaddapara T., Millar S.E. (2002). Wnt signals are required for the initiation of hair follicle development. Dev. Cell.

[26] Van Amerongen R., Bowman A.N., Nusse R. (2012). Developmental stage and time dictate the fate of Wnt/β-Catenin-responsive stem cells in the mammary gland. Cell Stem Cell.

[27] Barker N., van Es J.H., Kuipers J., Kujala P., van den Born M., Cozijnsen M., Haegebarth A., Korving J., Begthel H., Peters P.J. (2007). Identification of stem cells in small intestine and colon by marker gene *lgr5*. Nature.

[28] Hao H.X., Xie Y., Zhang Y., Charlat O., Oster E., Avello M., Lei H., Mickanin C., Liu D., Ruffner H. (2012). ZNRF3 promotes Wnt receptor turnover in an R-spondin-sensitive manner. Nature.

[29] De Lau W., Peng W.C., Gros P., Clevers H. (2014). The R-spondin/Lgr5/Rnf43 module: Regulator of Wnt signal strength. Genes Dev..

[30] Lim X., Tan S.H., Koh W.L., Chau R.M., Yan K.S., Kuo C.J., van Amerongen R., Klein A.M., Nusse R. (2013). Interfollicular epidermal stem cells self-renew via autocrine Wnt signaling. Science.

[31] Lyashenko N., Winter M., Migliorini D., Biechele T., Moon R.T., Hartmann C. (2011). Differential requirement for the dual functions of β-Catenin in embryonic stem cell self-renewal and germ layer formation. Nat. Cell Biol..

[32] Wray J., Kalkan T., Gomez-Lopez S., Eckardt D., Cook A., Kemler R., Smith A. (2011). Inhibition of glycogen synthase kinase-3 alleviates Tcf3 repression of the pluripotency network and increases embryonic stem cell resistance to differentiation. Nat. Cell Biol..

[33] Pereira L., Yi F., Merrill B.J. (2006). Repression of *NANOG* gene transcription by Tcf3 limits embryonic stem cell self-renewal. Mol. Cell. Biol..

[34] Yi F., Merrill B.J. (2010). Non-cell-autonomous stimulation of stem cell proliferation following ablation of Tcf3. Exp. Cell Res..

[35] Guo G., Huang Y., Humphreys P., Wang X., Smith A. (2011). A *Piggybac*-based recessive screening method to identify pluripotency regulators. PLoS ONE.

[36] Cole M.F., Johnstone S.E., Newman J.J., Kagey M.H., Young R.A. (2008). Tcf3 is an integral component of the core regulatory circuitry of embryonic stem cells. Genes Dev..

[37] Boyer L.A., Lee T.I., Cole M.F., Johnstone S.E., Levine S.S., Zucker J.P., Guenther M.G., Kumar R.M., Murray H.L., Jenner R.G. (2005). Core transcriptional regulatory circuitry in human embryonic stem cells. Cell.

[38] Loh Y.H., Wu Q., Chew J.L., Vega V.B., Zhang W., Chen X., Bourque G., George J., Leong B., Liu J. (2006). The Oct4 and NANOG transcription network regulates pluripotency in mouse embryonic stem cells. Nat. Genet..

[39] Marson A., Levine S.S., Cole M.F., Frampton G.M., Brambrink T., Johnstone S., Guenther M.G., Johnston W.K., Wernig M., Newman J. (2008). Connecting microRNA genes to the core transcriptional regulatory circuitry of embryonic stem cells. Cell.

[40] Tam W.L., Lim C.Y., Han J., Zhang J., Ang Y.S., Ng H.H., Yang H., Lim B. (2008). T-cell factor 3 regulates embryonic stem cell pluripotency and self-renewal by the transcriptional control of multiple lineage pathways. Stem Cells.

[41] Yi F., Pereira L., Merrill B.J. (2008). Tcf3 functions as a steady-state limiter of transcriptional programs of mouse embryonic stem cell self-renewal. Stem Cells.

[42] Takao Y., Yokota T., Koide H. (2007). β-Catenin up-regulates Nanog expression through interaction with Oct-3/4 in embryonic stem cells. Biochem. Biophys. Res. Commun..

[43] Kelly K.F., Ng D.Y., Jayakumaran G., Wood G.A., Koide H., Doble B.W. (2011). β-Catenin enhances Oct-4 activity and reinforces pluripotency through a Tcf-independent mechanism. Cell Stem Cell.

[44] Faunes F., Hayward P., Descalzo S.M., Chatterjee S.S., Balayo T., Trott J., Christoforou A., Ferrer-Vaquer A., Hadjantonakis A.K., Dasgupta R. (2013). A membrane-associated β-Catenin/Oct4 complex correlates with ground-state pluripotency in mouse embryonic stem cells. Development.

[45] Kielman M.F., Rindapaa M., Gaspar C., van Poppel N., Breukel C., van Leeuwen S., Taketo M.M., Roberts S., Smits R., Fodde R. (2002). APC modulates embryonic stem-cell differentiation by controlling the dosage of β-Catenin signaling. Nat. Genet..

[46] Doble B.W., Patel S., Wood G.A., Kockeritz L.K., Woodgett J.R. (2007). Functional redundancy of GSK-3α and GSK-3β in Wnt/β-Catenin signaling shown by using an allelic series of embryonic stem cell lines. Dev. Cell.

[47] Hao J., Li T.G., Qi X., Zhao D.F., Zhao G.Q. (2006). Wnt/β-Catenin pathway up-regulates *Stat3* and converges on LIF to prevent differentiation of mouse embryonic stem cells. Dev. Biol..

[48] Ogawa S., Tagawa Y., Kamiyoshi A., Suzuki A., Nakayama J., Hashikura Y., Miyagawa S. (2005). Crucial roles of mesodermal cell lineages in a murine embryonic stem cell-derived in vitro liver organogenesis system. Stem Cells.

[49] Lie D.C., Colamarino S.A., Song H.J., Desire L., Mira H., Consiglio A., Lein E.S., Jessberger S., Lansford H., Dearie A.R. (2005). Wnt signalling regulates adult hippocampal neurogenesis. Nature.

[50] Chenn A., Walsh C.A. (2002). Regulation of cerebral cortical size by control of cell cycle exit in neural precursors. Science.

[51] Zeng Y.A., Nusse R. (2010). Wnt proteins are self-renewal factors for mammary stem cells and promote their long-term expansion in culture. Cell Stem Cell.

[52] Wang D., Cai C., Dong X., Yu Q.C., Zhang X.O., Yang L., Zeng Y.A. (2015). Identification of multipotent mammary stem cells by protein C receptor expression. Nature.

[53] Huch M., Dorrell C., Boj S.F., van Es J.H., Li V.S., van de Wetering M., Sato T., Hamer K., Sasaki N., Finegold M.J. (2013). In vitro expansion of single Lgr5^+^ liver stem cells induced by Wnt-driven regeneration. Nature.

[54] Kohlmaier A., Edgar B.A. (2008). Proliferative control in *Drosophila* stem cells. Curr. Opin. Cell Biol..

[55] Yamashita Y.M., Fuller M.T., Jones D.L. (2005). Signaling in stem cell niches: Lessons from the *Drosophila* germline. J. Cell Sci..

[56] Mottier-Pavie V.I., Palacios V., Eliazer S., Scoggin S., Buszczak M. (2016). The Wnt pathway limits BMP signaling outside of the germline stem cell niche in *Drosophila* ovaries. Dev. Biol..

[57] Song X., Xie T. (2003). *Wingless* signaling regulates the maintenance of ovarian somatic stem cells in *Drosophila*. Development.

[58] Yamashita Y.M., Jones D.L., Fuller M.T. (2003). Orientation of asymmetric stem cell division by the APC tumor suppressor and centrosome. Science.

[59] Colosimo P.F., Tolwinski N.S. (2006). Wnt, Hedgehog and junctional armadillo/β-Catenin establish planar polarity in the *Drosophila* embryo. PLoS ONE.

[60] Kaplan N.A., Liu X., Tolwinski N.S. (2009). Epithelial polarity: Interactions between junctions and apical-basal machinery. Genetics.

[61] Kaplan N.A., Colosimo P.F., Liu X., Tolwinski N.S. (2011). Complex interactions between GSK3 and aPKC in *Drosophila* embryonic epithelial morphogenesis. PLoS ONE.

[62] Takashima S., Mkrtchyan M., Younossi-Hartenstein A., Merriam J.R., Hartenstein V. (2008). The behaviour of *Drosophila* adult hindgut stem cells is controlled by Wnt and Hh signalling. Nature.

[63] Clevers H., Loh K.M., Nusse R. (2014). Stem cell signaling. An integral program for tissue renewal and regeneration: Wnt signaling and stem cell control. Science.

[64] Tian A., Benchabane H., Wang Z., Zimmerman C., Xin N., Perochon J., Kalna G., Sansom O.J., Cheng C., Cordero J.B. (2017). Intestinal stem cell overproliferation resulting from inactivation of the APC tumor suppressor requires the transcription cofactors Earthbound and Erect wing. PLoS Genet..

[65] Nusse R., Clevers H. (2017). Wnt/β-Catenin signaling, disease, and emerging therapeutic modalities. Cell.

[66] Ghosh S., Tibbit C., Liu J.L. (2016). Effective knockdown of *Drosophila* long non-coding RNAs by CRISPR interference. Nucleic Acids Res..

[67] Chintapalli V.R., Wang J., Dow J.A. (2007). Using flyatlas to identify better *Drosophila melanogaster* models of human disease. Nat. Genet..

[68] Huisken J., Stainier D.Y. (2009). Selective plane illumination microscopy techniques in developmental biology. Development.

[69] Keller P.J., Schmidt A.D., Wittbrodt J., Stelzer E.H. (2011). Digital scanned laser light-sheet fluorescence microscopy (DSLM) of Zebrafish and *Drosophila* embryonic development. Cold Spring Harb. Protoc..

[70] Kaur P., Saunders T.E., Tolwinski N.S. (2017). Coupling optogenetics and light-sheet microscopy, a method to study Wnt signaling during embryogenesis. Sci. Rep..

[71] Johnson H.E., Goyal Y., Pannucci N.L., Schupbach T., Shvartsman S.Y., Toettcher J.E. (2017). The spatiotemporal limits of developmental ERK signaling. Dev. Cell.

[72] Huang A., Amourda C., Zhang S., Tolwinski N.S., Saunders T.E. (2017). Decoding temporal interpretation of the morphogen bicoid in the early *Drosophila* embryo. Elife.

[73] Masui S., Nakatake Y., Toyooka Y., Shimosato D., Yagi R., Takahashi K., Okochi H., Okuda A., Matoba R., Sharov A.A. (2007). Pluripotency governed by SOX2 via regulation of Oct3/4 expression in mouse embryonic stem cells. Nat. Cell Biol..

[74] Huggins I.J., Bos T., Gaylord O., Jessen C., Lonquich B., Puranen A., Richter J., Rossdam C., Brafman D., Gaasterland T. (2017). The WNT target SP5 negatively regulates WNT transcriptional programs in human pluripotent stem cells. Nat. Commun..

[75] Suresh J., Harmston N., Lim K.K., Kaur P., Jin H.J., Lusk J.B., Petretto E., Tolwinski N.S. (2017). An embryonic system to assess direct and indirect Wnt transcriptional targets. Sci. Rep..

[76] Franz A., Shlyueva D., Brunner E., Stark A., Basler K. (2017). Probing the canonicity of the Wnt/Wingless signaling pathway. PLoS Genet..

[77] Technau G.M., Campos-Ortega J.A. (1987). Cell autonomy of expression of neurogenic genes of *Drosophila melanogaster*. Proc. Natl. Acad. Sci. USA.

[78] Tulina N., Matunis E. (2001). Control of stem cell self-renewal in *Drosophila* spermatogenesis by JAK-STAT signaling. Science.

[79] Ohlstein B., Spradling A. (2006). The adult *Drosophila* posterior midgut is maintained by pluripotent stem cells. Nature.

[80] Kaur P., Tolwinski N. (2015). *Myc*, *luna*, *nubbin* and *sox21a* appear to be the closest homologues of pluripotency genes in *Drosophila* based on sequence comparison.

[81] Li M., Izpisua Belmonte J.C. (2016). Looking to the future following 10 years of induced pluripotent stem cell technologies. Nat. Protoc..

[82] Ocampo A., Reddy P., Martinez-Redondo P., Platero-Luengo A., Hatanaka F., Hishida T., Li M., Lam D., Kurita M., Beyret E. (2016). In vivo amelioration of age-associated hallmarks by partial reprogramming. Cell.

[83] Wu J., Ocampo A., Belmonte J.C.I. (2016). Cellular metabolism and induced pluripotency. Cell.

[84] Gruber J., Yee Z., Tolwinski N.S. (2016). Developmental drift and the role of Wnt signaling in aging. Cancers (Basel).

